# Circulating tumor cells in patients undergoing androgen deprivation therapy with versus without cryosurgery for metastatic prostate cancer: a retrospective analysis

**DOI:** 10.1186/s12957-021-02455-4

**Published:** 2021-12-13

**Authors:** Mingxiong Sheng, Shanming Guo, Chunxiao Liu

**Affiliations:** 1 Department of Urology, Zhujiang Hospital, Southern Medical University, No. 253, Industrial Rd., Guangzhou, Guangdong Province 510282 People’s Republic of China; 2 Department of Urology, Mindong Hospital affiliated to Fujian Medical University, Fuan, Fujian Province 355000 People’s Republic of China

**Keywords:** Prostate cancer, Cryosurgery, Androgen deprivation therapy, Circulating tumor cell

## Abstract

**Background:**

The study aimed to assess the value of circulating tumor cells (CTCs) as a prognostic and treatment response marker in patients undergoing androgen deprivation therapy (ADT) plus cryosurgery vs. ADT alone for metastatic prostate cancer (mPCA).

**Methods:**

This retrospective analysis included 43 patients with mPCA: 23 receiving ADT alone (control) and 20 receiving additional cryosurgery (cryosurgery group). CTCs and progression-free survival (PFS) were compared between the two groups. Cox proportional hazards regression was conducted to identify variables associated with PFS.

**Results:**

Median PFS was 35 months (IQR, 33‑37) in the cryosurgery group vs. 30 months (IQR, 27‑32) in the control (*p* < 0.001). CTCs count was significantly lower in the cryosurgery group at both 3 months (*z* = 2.170, *p* = 0.030) and 12 months (*z* = 2.481; *p* = 0.013). In comparison to the baseline, the number of CTCs at both 3 and 12 months was lower in the cryosurgery group (*p* = 0.004 and *p* < 0.001, respectively), but not in the ADT alone group. In multivariate Cox regression, shorter PFS was associated with baseline PSA ≧100 ng/ml (HR 6.584, 95% CI, 5.309‑8.166), biopsy Gleason score ≧ 8 (HR 2.064, 95% CI, 1.608‑2.650), clinic T stage>T2b (HR 5.021, 95% CI, 3.925‑6.421), number of bone metastases>3 (HR 3.421, 95% CI, 2.786‑4.202), positive CTCs at 3 months post-treatment (HR 6.833, 95% CI, 5.176‑9.022), positive CTCs 1 year post-treatment (HR 6.051, 95% CI, 4.347‑8.424). Prostate cryosurgery was associated with longer PFS (HR 0.062, 95% CI, 0.048‑.080).

**Conclusions:**

CTC was a prognostic and treatment response marker for mPCA. ADT plus cryosurgery could reduce CTCs and prolong PFS vs. ADT alone in mPCA patients with low metastatic volume.

## Introduction

Androgen deprivation therapy (ADT) is the standard treatment for metastatic prostate cancer (mPCA). Previous studies showed that patients with mPCA could benefit from treatment of the primary tumor, including survival benefit or reduced incidence of local complications [1, 2]. The STAMPED trial [3] demonstrated improved overall survival (OS) with ADT plus prostate radiotherapy (RT) to the primary tumor vs. ADT alone only in patients with low-volume disease, but not in patients with high-volume disease. In a retrospective study, Si et al. [4] reported significant benefits in both OS and progress-free survival (PFS) in mPCA patients receiving cryosurgery plus ADT than ADT alone. However, there is no reliable marker to monitor treatment response (sensitivity to cryosurgery or ADT). The primary tumor could be a source of circulating tumor cells (CTCs); radical resection or intensified radiotherapy of the primary tumor in mPCA patients reduced the source of CTCs and improved the survival [5]. Recent studies indicate CTC count could predict the prognosis in patient with metastatic castration-resistant prostate cancer (mCRPC) [6–10]. Two prospective trials with abiraterone and chemotherapy [10, 11] showed that changes in CTCs as early as 4 weeks after treatment could identify patients who were not benefiting from treatment. The finding suggested that the CTC count could be an intermediate biomarker for overall survival in advanced mPCA. Up to date, there has been no study on CTCs in patients undergoing cryosurgery for mPCA. Therefore, the value of CTCs as a prognostic marker in patients undergoing cryosurgery for mPCA remains unclear. We hypothesized CTC count could identify patients with a poorer prognosis after treatment (cryosurgery or ADT) and predict treatment response in patients with mPCA. We conducted a retrospective study to compare CTC count and PFS in patients with mPCA receiving ADT plus cryosurgery versus ADT alone. The manuscript was prepared in accordance with the STROBE reporting checklist.

## Methods

### Patients

This study complied with the tenets of the Declaration of Helsinki (as was revised in 2013), and was approved by the Ethics Committee of Mindong Hospital Affiliated to Fujian Medical University on December 18, 2017 (Number #0518-4). All patients undergoing either ADT plus cryosurgery (referred to as cryosurgery group below) or ADT alone (referred to as control group below) as initial therapy for prostate cancer with bone metastasis but no visceral metastasis at our clinic during a period from January 2015 to September 2017 were included in the retrospective analysis. Cryosurgery was recommended to all patients. The treatment (ADT alone vs. ADT plus cryosurgery) was based on patient choice. The criteria for inclusion in the analysis included (1) prostate cancer was confirmed by needle biopsy; (2) bone metastases were detected by nuclide bone scan and without super bone scan; (3) no visceral metastasis; (4) T stage ≤ cT3a; (5) presence of CTCs in peripheral blood before treatment, as defined by ≧ 2 CTCs in 5-ml blood using a CTC capture instrument NEXT CTC FS008 (NaoBio, China); (6) no previous local therapy; (7) prostate volume ≤ 50 ml. Informed consent was provided by either the patient or family member. The exclusion criteria included (1) emergence of mCRPC within 6 months after ADT. (2) Specific pathological types of prostate cancer other than acinar adenocarcinoma, including sarcoma, small cell carcinoma, and transitional cell carcinoma.

ADT consisted of 50-mg bicalutamide per day and subcutaneous injection of 3.75-mg leuprolide every month. Cryosurgery was performed by the same surgeon using the CRYO care system (Endocare Incorporated, USA). Briefly, patients were placed in a dorsal lithotomy position and received local infiltration anesthesia with 10-ml 1% lidocaine. A urethral warming catheter was inserted into bladder to protect the urethra. 17-G cryoprobes were inserted under transrectal ultrasonography guidance and spaced approximately 1.0 cm apart. The cryoprobes were placed according to prostate size and shape, so that the entire prostatic gland was covered. Warm saline irrigation was conducted continuously to avoid urethral freezing. Two freeze-thaw cycles were conducted. Each freezing cycle lasted for 10‑15 min. After surgery, the urethral warming unit was kept in place for 5 min. The Foley catheter was removed 3 weeks later.

### Follow-up

The follow-up was conducted every month during the first year, and every 3 months thereafter, and consisted of serum prostate specific antigen (PSA), testosterone, standard liver and kidney functional tests, and rectal digital examination. Prostate magnetic resonance imaging and nuclide bone scan were performed if progression (either biochemical or clinical) was suspected.

### Outcome measures

Outcome measures included (1) demographics (age and gender) and clinical characteristics (prostate volume, PSA, biopsy Gleason score, clinical stage, and bone metastases count); (2) PFS; (3) CTC counts.

PFS was defined as the time from the initiation of ADT to the first evidence of biochemical or radiological progression. Biochemical progression was defined as three consecutive rises in PSA at least 1 week apart resulting in two 50% increases over the nadir, and a PSA > 2 ng/ml. Radiological progression was defined as the appearance of new lesions: either two or more new bone lesions on bone scan or a soft tissue lesion using RECIST (response evaluation criteria in solid tumors). Symptomatic progression alone must be questioned and subject to further investigation.

CTCs were detected using a CTC capture instrument NEXT CTC FS008 (NaoBio Company, China) at baseline, 3 months and 12 months after treatment. Five milliliters of peripheral blood samples was collected and pretreated. CTCs were captured by Next CTC FS008 based on Nano microfluidics [12, 13]. Cell sorting utilizes the physical characteristics of large nucleocytoplasmic ratio and negative charge on the surface of CTCs. The captured cells were identified as CTCs by fluorescence in situ hybridization (FISH). Cells with the signal in nucleus ≥ 3 triploid, positive DAPI, and negative CD45 were considered CTCs.

### Statistical analysis

Continuous variables were analyzed with Student’s *t* test for independent samples. Categorical variables were analyzed using chi square test. Ranked variables were analyzed using the Wilcoxon rank sum test. PFS was analyzed using Kaplan-Meier analysis followed by a log-rank test. Cox proportional hazards regression was conducted to identify variables associated with PFS. In addition to the main analysis, we conducted subgroup analyses based on metastatic volume. High metastatic volume was defined as ≥ 4 bone metastatic lesions that included at least 1 outside the vertebral column [5]; the others were considered as low metastatic volume. Statistical significance was defined as *p* < 0.05 (2-sided). All analyses were performed using the SPSS20.0 statistical software ((IBM Corp., Armonk, NY, USA).

## Results

### Baseline patient characteristics

A total of 43 patients were included in the analysis. Twenty patients received cryosurgery plus ADT, and the remaining 23 received ADT only. Mean age was 70.3 ± 7.5 years (range, 55‑83) in the cryosurgery group and 68.7 ± 7.2 years (range, 56‑84) in the control group (*p* = 0.479; Table 1). The 2 groups did not differ significantly in serum PSA, biopsy Gleason score, clinical stage, and bone metastases count. Median CTC count was 4 (IQR, 3‑6) in the cryosurgery group and 6 (IQR, 3‑7) in the control group (*p* = 0.475).

**Table 1:** Demographic and baseline characteristics

	ADT alone, *n* = 23	ADT + cryosurgery, *n* = 20	*p*
Age (years), mean ± SD	68.7 ± 7.2	70.3 ± 7.5	0.479
≤60, *n* (%)	3 (13.0%)	2 (10%)	0.716
60‑70, *n* (%)	14 (60.9%)	12 (60%)	
> 70, n (%)	6 (26.1%)	6 (30%)	
PSA (ng/ml), mean ± SD	84.16 ± 27.45	84.83 ± 31.77	0.074
Biopsy Gleason score, *n* (%)			
≤ 6	1 (4.3%)	2 (10%)	0.615
7	8 (34.8%)	7 (35%)	
≥ 8	14 (60.9%)	11 (55%)	
Prostate volume (ml), mean ± SD	42.00 ± 3.73	42.30 ± 4.24	0.806
Clinical T stage, *n* (%)			
≤cT2b	8 (34.8%)	5 (25%)	0.555
cT2c	10 (43.5%)	10 (50%)	
cT3a	5 (21.7%)	5 (25%)	
Lymph nodes status, *n* (%)			
N0	18 (78.3%)	15 (75%)	0.801
N1	5 (21.7%)	5 (25%)	
Bone metastases count, *n* (%)			
≤ 3	10 (43.5%)	11 (55%)	0.451
> 3	13 (56.5%)	9 (45%)	
CTCs count, median (IQR)	6 (3‑7)	4 (3‑6)	0.475

*ADT* androgen deprivation therapy, *CTC* circulating tumor cell, *IQR* interquartile range, *PSA* prostate specific antigen, *SD* standard deviation

### Complications after cryosurgery

Surgery was completed in all 20 patients who opted to receive cryosurgery. The average operative time was 93.3 ± 12.1 min. Urinary tract infection was noted in 7 (35%) patients, but dissipated by empirical antibiotic therapy. No urinary incontinence, urethra injury, rectal injury, and recto-urethral fistulas occurred. All patients could urinate at will after removing the catheter at 3 weeks after surgery.

### Oncological outcomes

**Fig. 1:**
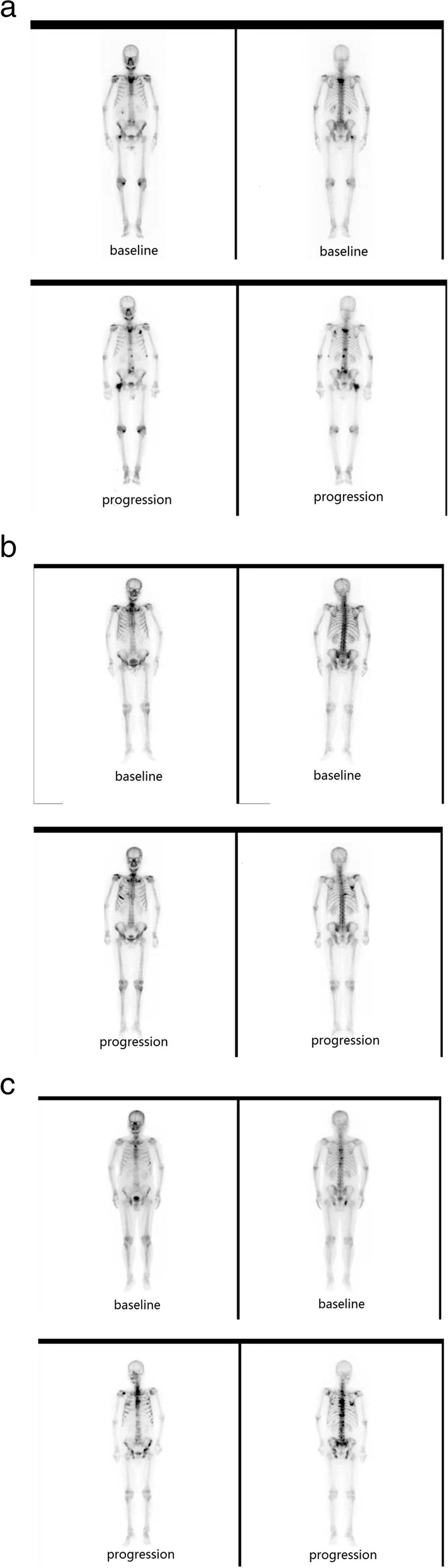
**A** Nuclide bone scan image of case 1 in the control: baseline vs. radiological progression. **B** Nuclide bone scan image of case 2 in the control: baseline vs. radiological progression. **C** Nuclide bone scan image of case 1 in the ADT plus cryosurgery group: baseline vs. radiological progression

The median follow-up was 32 months (range, 22‑36) in the control group and 36 months (range, 26‑38) in the cryosurgery group. During the follow-up period, two (8.70%) patients in the control group died of myocardial infarction; two patients developed only radiological progression (Fig. 1A, B), and 19 patients developed PSA progression. In terms of subsequent therapy in patients with mCRPC (*n* = 21), 5 patients (23.8%) had no second-line treatment, 10 patients (47.6%) received abiraterone, and 6 patients (28.6%) received chemotherapy with docetaxel. In the cryosurgery group, two (10%) patients died of myocardial infarction and 1 (5%) patient died of stroke; one patient developed only radiological progression (Fig. 1C), and 16 patients developed PSA progression. In terms of subsequent therapy in patients with mCRPC (*n* = 17), 3 (17.6%) patients had no second-line treatment, 9 (52.9%) patients received abiraterone and 5 (29.4%) patients received chemotherapy with docetaxel.

**Fig. 2:**
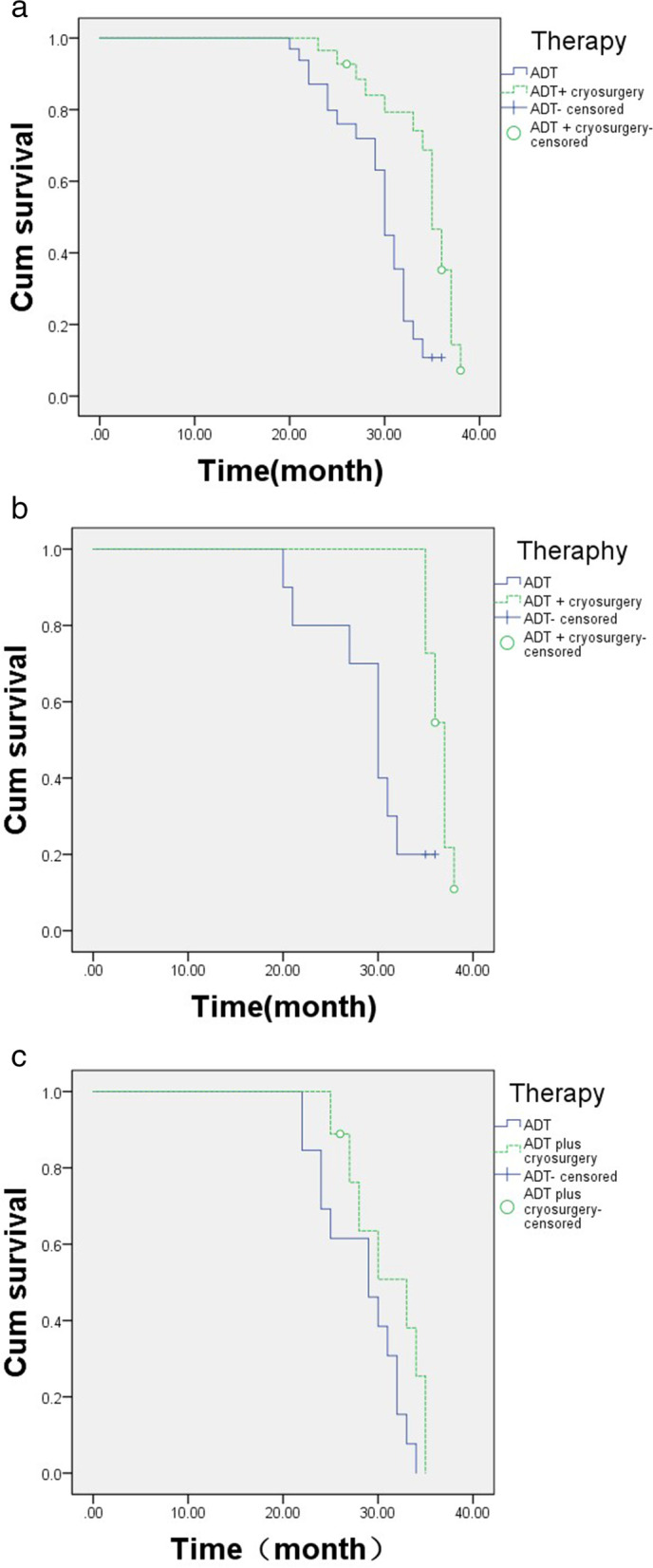
**A** Progression-free survival: a comparison between ADT plus cryosurgery (*n* = 20) vs. ADT alone (*n* =2 3). **B** Sub-group analysis of progression-free survival in the patients with low metastatic volume: a comparison between ADT plus cryosurgery (*n* = 11) vs. ADT alone (*n* = 10). **C** Sub-group analysis of progression-free survival in the patients with high metastatic volume: a comparison between ADT plus cryosurgery (*n* = 9) vs. ADT alone (*n* = 13)

The median PFS was 35 months [interquartile range (IQR) 33‑37] in the cryosurgery group vs. 30 months (IQR 27‑32) in the control group (*p* < 0.001, Fig. 2A). In the multivariate Cox regression, shorter PFS was independently associated with the following variables: baseline PSA ≥ 100 ng/ml [hazard rate (HR) 6.584, 95% CI, 5.309‑8.166], biopsy Gleason score ≧ 8 (HR 2.064, 95% CI, 1.608‑2.650), clinic T stage > T2b (HR 5.021, 95% CI, 3.925‑6.421), number of bone metastases > 3 (HR 3.421, 95% CI, 2.786‑4.202), positive CTCs at 3 months post-treatment (HR 6.833, 95% CI, 5.176‑9.022), positive CTCs 1 year post-treatment (HR 6.051, 95% CI, 4.347‑8.424). Prostate cryosurgery was associated with longer PFS (HR 0.062, 95% CI, 0.048‑.080) (Table 2).

**Table 2:** Multivariate Cox regression of progression-free survival

Variable	Hazards ratio	95% CI	*P* value
Prostate cryosurgery (vs. ADT alone)	0.062	0.048‑0.080	< 0.001
PSA ≥100 ng/ml (vs.< 100ng/ml)	6.584	5.309‑8.166	< 0.001
Gleason score ≥ 8 (vs.< 8)	2.064	1.608‑2.650	< 0.001
Clinical T stage > T2b (vs. ≤ T2b)	5.021	3.925‑6.421	< 0.001
Bone metastases count > 3 (vs.≤ 3)	3.421	2.786‑4.202	< 0.001
Positive CTCs at 3-month (vs. negative)	6.833	5.176‑9.022	< 0.001
Positive CTCs at 1-year (vs. negative)	6.051	4.347‑8.424	< 0.001

*CI* confidence interval, *CTC* circulating tumor cell, *PSA* prostate specific antigen

### CTC count

CTC count was significantly lower in the cryosurgery group at both 3 months (*z* = 2.170, *p* = 0.030) and 12 months (*z* = 2.481; *p* = 0.013). The percentage of patients with > 5 CTCs per 5-ml peripheral blood was lower in the cryosurgery group at both 3 (15.5% vs. 43.5%, *p* = 0.030) and 12 months (0 vs. 30.4%, *p* = 0.013) (Table 3). In comparison to the baseline, the number of CTCs at both 3 and 12 months was lower in the cryosurgery group (*p* = 0.004 and *p* < 0.001, respectively), but not in the ADT alone group.

**Table 3:** Number/percentage of the patients with different number of CTCs count at baseline, 3 months, and 12 months after treatment

	ADT alone, *n* = 23	ADT + cryosurgery, *n* = 20	*Z* value	*P* value
Baseline, *n* (%)				
< 2	0 (0%)	0 (0%)	0.789	0.430
2‑5	11 (47.8%)	12 (60%)		
≥5	12 (52.2%)	8 (40%)		
3 months, *n* (%)				
< 2	4 (17.4%)	8 (40%)	2.170	0.030
2‑5	9 (39.1%)	9 (45%)		
≥ 5	10 (43.5%)	3 (15%)		
12 months, *n* (%)				
< 2	9 (39.1%)	14 (70%)	2.481	0.013
2‑5	7 (30.4%)	6 (30%)		
≥ 5	7 (30.4%)	0 (0%)		

*ADT* androgen deprivation therapy

### Subgroup analysis

Among the 43 patients, 21 patients had low metastatic volume, and 22 patients had high metastatic volume. In the 21 patients with low metastatic volume, 11 received ADT plus cryosurgery group, and the remaining 10 received ADT alone. In the 22 patients with high metastatic volume, 9 patients received ADT plus cryosurgery, and the remaining 13 received ADT alone. Median baseline bone metastases count was 2 (IQR 1‑3) vs. 8 (IQR 6‑11) in patients with low vs. high metastatic volume (*p* < 0.001). Median baseline CTC count was 3 (IQR 3‑4) vs. 7 (IQR 6‑8) in patients with low vs. high metastatic volume (*p* < 0.001).

In the subgroup analysis that included the 21 patients with low metastatic volume only, CTC count was significantly lower in the cryosurgery group at both 3 months (*z* = 1.969, *p* = 0.049) and 12 months (*z* = 2.622, *p* = 0.009) (Table 4). The median PFS was 37 months (IQR 35‑37) in cryosurgery vs. 30 months (IQR 27‑32) in the control group (*p* = 0.004; Fig. 2B).

**Table 4:** Sub-group analysis of CTC count in patients with low metastatic volume

	ADT alone, *n* = 10	ADT + cryosurgery, *n* = 11	*Z* value	*P* value
Baseline, *n* (%)				
<2	0 (0%)	0 (0%)	0.000	1.000
2-5	10 (100%)	11 (100%)		
≥5	0 (0%)	0 (0%)		
3 months, *n* (%)				
<2	2 (20%)	7 (63.6%)	1.969	0.049
2-5	8 (80%)	4 (36.4%)		
≥5	0 (0%)	0 (0%)		
12 months, *n* (%)				
< 2	5 (50%)	11 (100%)	2.622	0.009
2‑5	5 (50%)	0 (0%)		
≥ 5	0 (0%)	0 (0%)		

*ADT* androgen deprivation therapy

In the subgroup analysis that included the 22 patients with high metastatic volume only, CTC count did not differ between the 2 groups at any time point (Table 5). The median PFS was 33 months (IQR 28‑35) in cryosurgery vs. 29 months (IQR 24‑32) in the control group (*p* = 0.076; Fig. 2C).

**Table 5:** Sub-group analysis of CTCs count in patients with high metastatic volume

	ADT alone, *n* = 13	ADT + cryosurgery, *n* = 9	*Z* value	*P* value
Baseline, *n* (%)				
< 2	0 (0%)	0 (0%)	0.000	1.000
2‑5	0 (0%)	0 (0%)		
≥ 5	13 (100%)	9 (100%)		
3 months, *n* (%)				
< 2	2 (15.4%)	1 (11.1%)	1.596	0.111
2‑5	1 (7.7%)	5 (55.6%)		
≥ 5	10 (76.9%)	3 (33.3%)		
12 months, *n* (%)				
< 2	4 (30.8%)	3 (33.3%)	1.593	0.111
2‑5	2 (15.4%)	6 (66.7%)		
≥ 5	7 (53.8%)	0 (0%)		

*ADT* androgen deprivation therapy

## Discussion

Consistent with a previous study by Si et al. [4], the current study demonstrated longer PFS in the patients receiving ADT plus surgery for mPCA than ADT alone. Of note, we found survival benefit for ADT plus cryosurgery only in patients with low metastatic volume, but not high metastatic volume. In the STAMPEDE trial [3], radiotherapy to the primary tumor did not improve OS in the overall study population. In the subgroup analysis that divided the patients based on the CHAARTED standard, however, radiotherapy increased the OS in the low-volume subgroup. Results in subgroup analysis in our study were consistent with the STAMPED study.

Cox multivariate analysis showed that the patients with positive CTCs at 3 months or 1 year post-treatment had shorter PFS. In the IMMC38 trial, de Bono et al. [7] detected CTCs in 231 CRPC patients receiving chemotherapy with CellSearch system, and showed a close association between shorter OS with higher CTC at both prior to and after treatment. Mandel et al. [14] examined CTCs in 33 patients with hormone-naïve oligometastatic prostate cancer undergoing cytoreductive radical prostatectomy, and found earlier CRPC and shorter OS in patients with CTCs ≥ 2 in 7.5-ml peripheral blood at 6 months postoperatively. The results suggest that high CTC is a risk for poor prognosis, and maybe helpful in selecting hormone-naïve oligometastatic prostate cancer patients for cytoreductive radical prostatectomy. The patients with lower CTCs were suitable candidate for cytoreductive radical prostatectomy. To our best knowledge, this is the first study which found CTC count could identify patients with a poorer prognosis after treatment (cryosurgery or ADT). The patients with positive CTCs at 3 months or 1 year post-treatment had poorer prognosis after treatment (cryosurgery or ADT).

Our study also showed CTC count could determine the degree of response to the treatment. In the current study, the patients receiving ADT plus cryosurgery had lower CTCs at 3 months and 1 year after treatment, and longer PFS than those receiving ADT alone. In comparison to the baseline, the number of CTCs at both 3 and 12 months was lower in the cryosurgery group, but not in the ADT alone group. Lower CTCs in the patients receiving ADT plus cryosurgery was evident in the subgroup analysis that included patients with low metastatic volume only, but not in the subgroup that included the patients with high metastatic volume. Such a finding is consistent with the survival benefit in patients with low metastatic volume only. These results suggested in the patients with low metastatic volume, those receiving ADT plus cryosurgery had lower CTCs and responded better to the treatment than those receiving ADT alone. No similar effect was found in the patients with high metastatic volume.

The higher number of CTCs might be associated with advanced clinical stage and higher metastatic volume [15]. The positive rate of CTCs detected by CellSearch system was 0‑10% in healthy volunteers [16, 17], 5‑38.4% in non-metastatic high risk prostate cancer patients [16–19], 48.5% in oligometastatic prostate cancer [14], and 80% in patients with mCRPC [20]. In our study, the number of CTCs was significantly higher in the patients with high metastatic volume. Because CTCs most likely represent an aggressive pool of cells with the potential to form metastases, the patient with higher CTCs might develop more bone metastases. The patients with high bone metastases might have more source of CTCs and have higher CTCs. But our hypothesis still needs to be tested and the mechanism remains unclear. There was limited study to assess the association of CTC counts with bone metastases volume. In a previous study of 19 metastatic patients with only bone metastases (mCRPC and metastatic taxane-refractory) [17], no correlation was observed between CTC counts and osseous tumor burden assessed by bone lesion count or bone scan index. Such a difference may reflect different patient characteristics (hormone-sensitive in the current study vs. mCRPC and metastatic taxane-refractory in previous study).

CTCs could derive from either primary tumor or metastatic foci. We hypothesize that since primary tumor represents the major source of CTCs in patients with low metastatic volume, resection of the primary tumor could decrease the number of CTCs, and could be helpful in disease control. In contrast, contribution of the primary tumor to CTCs is relatively small. As a result, the impact of local treatment for primary tumor on patient survival is minimal.

Satkunasivam et al. [5] summarized the mechanisms of survival benefits from local therapy (radical prostatectomy or radiotherapy). First, eradication of the primary tumor eliminates the source of cytokine signaling that prepares niches for eventual sites of metastases and promotes their growth [21]. Second, the primary tumor may remain a source of CTCs that are capable of “self-seeding” the primary organ [22]. Third, local therapy may reduce the number of self-renewing cells that persist after ADT due to the low levels of immature androgen receptors [23]. Local treatment against the primary tumor may induce inflammatory reaction and promote antigen production, which in turn could induce anti-tumor immune responses. Therefore, removal of the primary tumor may reduce metastases [23, 24]. Cryosurgery ablates tumors in situ, leading to the release of tumor proteins and intact tumor associated antigens. Residual tumor antigens may activate anti-tumor immune response in the inflammatory microenvironment [25]. Our current study confirmed that prostate cryosurgery could reduce the source and number of CTCs and then prolong PFS.

The current study has several limitations. As a retrospective analysis, this study has inherent bias in patient selection. Second, small sample size is an important limitation in the current study. As such, the results must be considered preliminary despite of the statistically significant differences between the two groups. Third, the follow-up was relatively short; we therefore did not analyze cancer-specific survival and OS.

## Conclusions

In summary, CTC was a prognostic and treatment response marker in patients undergoing androgen deprivation therapy (ADT) plus cryosurgery or ADT alone for mPCA. ADT plus cryosurgery could reduce CTCs and prolong PFS vs. ADT alone in mPCA patients with low metastatic volume.

## Data Availability

The datasets used and/or analyzed during the current study are available from the corresponding author on reasonable request.

## Abbreviations

ADT: Androgen deprivation therapy
mPCA: Metastatic prostate cancer
OS: Overall survival
RT: Radiotherapy
PFS: Progress-free survival
CTCs: Circulating tumor cells
mCRPC: Metastatic castration-resistant prostate cancer
PSA: Prostate specific antigen
IQR: Interquartile range
HR: Hazard rate
